# SARS-CoV-2 transmission in educational settings during an early summer epidemic wave in Luxembourg, 2020

**DOI:** 10.1186/s12879-021-06089-5

**Published:** 2021-05-04

**Authors:** Joël Mossong, Laurent Mombaerts, Lisa Veiber, Jessica Pastore, Gwenaëlle Le Coroller, Michael Schnell, Silvana Masi, Laetitia Huiart, Paul Wilmes

**Affiliations:** 1 Health Directorate, 1A-G Route de Trèves, L-2632 Findel, Luxembourg, Luxembourg; 2 Luxembourg Centre for Systems Biomedicine, University of Luxembourg, Esch-sur-Alzette and Belvaux, Luxembourg; 3 Interdisciplinary Centre for Security, Reliability and Trust, University of Luxembourg, Esch-sur-Alzette and Belvaux, Luxembourg; 4 Department of Population Health, Luxembourg Institute of Health, Luxembourg, Luxembourg; 5 Department of Life Sciences and Medicine, Faculty of Science, Technology and Medicine, University of Luxembourg, Belvaux, Luxembourg

## Abstract

**Background:**

Following a first wave in spring and gradual easing of lockdown, Luxembourg experienced an early second epidemic wave of SARS-CoV-2 before the start of summer school holidays on 15th July. This provided the opportunity to investigate the role of school-age children and school settings for transmission.

**Methods:**

We compared the incidence of SARS-CoV-2 in school-age children, teachers and the general working population in Luxembourg during two epidemic waves: a spring wave from March–April 2020 corresponding to general lockdown with schools being closed and May–July 2020 corresponding to schools being open. We assessed the number of secondary transmissions occurring in schools between May and July 2020 using routine contact tracing data.

**Results:**

During the first wave in March–April 2020 when schools were closed, the incidence in pupils peaked at 28 per 100,000, while during the second wave in May–July 2020 when schools were open, incidence peaked 100 per 100,000. While incidence of SARS-CoV-2 was higher in adults than in children during the first spring wave, no significant difference was observed during the second wave in early summer. Between May and July 2020, we identified a total of 390 and 34 confirmed COVID-19 cases among 90,150 school-age children and 11,667 teachers, respectively. We further estimate that 179 primary cases caused 49 secondary cases in schools. While some small clusters of mainly student-to-student transmission within the same class were identified, we did not observe any large outbreaks with multiple generations of infection.

**Conclusions:**

Transmission of SARS-CoV-2 within Luxembourg schools was limited during an early summer epidemic wave in 2020. Precautionary measures including physical distancing as well as easy access to testing, systematic contact tracing appears to have been successful in mitigating transmission within educational settings.

**Supplementary Information:**

The online version contains supplementary material available at 10.1186/s12879-021-06089-5.

## Introduction

While several reports indicate a limited role for children in transmission COVID-19 [1], epidemiological data of SARS-CoV-2 transmission in educational settings is scarce [2–4]. By threatening their social and mental wellbeing, it is becoming increasingly clear that closing schools poses also a risk for children’ educational needs [1]. Moreover, high economic repercussions including parents being more likely to take time off to care for children [5] mean that school closures should be considered a public health measure of last resort [1, 6].

Following a gradual easing of lockdown measures after the first spring epidemic wave in March–April, unlike most other countries in Europe, Luxembourg experienced an early second summer wave of COVID-19 infections before the start of summer holidays. Whereas schools were closed during the lockdown in spring, the early resurgence of cases during the second wave provided a unique opportunity to investigate the role of schools and children in relation to overall transmission in the wider community.

We performed an epidemiological analysis of COVID-19 cases in Luxembourg by comparing the incidence in school-age children and teachers to that of the general working population prior to the summer holidays 2020. Further, we estimated the number of secondary transmissions occurring at schools during the second wave using data from routine infectious disease surveillance data and contact tracing organized by the Directorate of Health.

## Methods

Following a rapid increase of SARS-CoV-2 cases in early March 2020, at which time no non-pharmaceutical measures or social distancing were in place, all primary and secondary schools were closed on 18th March (week 12) starting a general lockdown period. Schools reopened gradually in May: final year classes and other classes of secondary schools resumed on 4th May (week 19) and May 11th (week 20), respectively. Primary schools resumed 25th May (week 22), with the class size reduced by a factor of 2 and by alternating half of the classes each week, to be able to respect a minimal distance of 2 m between pupils. From 29th June (week 27) to 15th July (week 29), alternating classes were reunited and class size was normal again, i.e. maximum of 29 pupils in class for a normal school regimen and a maximum of 19 pupils for a class with children with special needs. While no wearing of masks was recommended before the lockdown, following the re-opening of schools in May, face masks became mandatory for children 6 years or older when the distance of 2 m could not be respected, i.e. during school transport, during breaks and when moving between classrooms. Once sitting down inside the classroom, face masks could be removed during lessons. No sports or social activities were organized in the schools and school canteens were closed.

To accompany the progressive lifting of lockdown measures, a mass screening programme was started in Luxembourg in May aiming to provide additional testing capacity for screening asymptomatic cases in certain working sectors including pupils and teachers [7]. Briefly, all teachers and pupils received an invitation for a PCR using a throat swab in dedicated Covid-19 screening sites. Between May and July, 33,723 and 14,657 tests were conducted among pupils and teachers, respectively, yielding 31 (0.09%) and 5 (0.03%) positive results, respectively. In addition to the mass screening, persons with symptoms seeking care were able to get free PCR (polymerase chain reaction)-tests in clinical laboratories with a prescription from a medical doctor, in dedicated COVID-19 treatment centres or in hospitals.

This analysis pertains to all confirmed positive cases of SARS-CoV-2 detected by PCR reported on a mandatory basis by clinical laboratories and which were automatically included in the contact tracing management system of the Health Directorate. Once a new positive result was reported via secure electronic reporting to the Health Directorate, the index case was contacted by phone usually on the same day. Positive cases were asked to self-isolate immediately and take precautions to avoid contact with other household members. Then, all high-risk contacts occurring within 48 h before symptom onset (or before date of test if asymptomatic) were contacted to self-quarantine. A contact was considered high risk if there was physical contact or close proximity (< 2 m) to a case for at least 15 min without wearing a mask. In accordance with national recommendations, for each quarantined contact, a laboratory test was automatically prescribed on the 5th day after the date of last contact. Contacts were instructed to test earlier if they became symptomatic before the assigned test date. If the test was negative, the quarantine ended automatically on the 8th day after the date of last contact and was followed by 7 days of self-surveillance; if it was positive, the person was contacted again by the contact tracing team as a new positive case starting the contact tracing procedure anew. If the contact did not take a test before the 7th day, the period of quarantine was automatically extended by 7 days to a total of 14 days.

When a confirmed cases had high-risk contacts at school, all pupils within the same class as a primary case were systematically quarantined. This measure was also applied to teachers and educators in contact with the primary case, if a high-risk exposure (> 15 min, < 2 m, incorrect mask use) was reported. We put in place a formal collaboration with the Ministry of Education and therefore the respective schools to rapidly identify all high-risk contacts usually within the same day as the positive result was reported. This information was transferred to the contact tracing team who was in charge of implementing tests and quarantine policy.

The COVID-19 surveillance data was linked to the national database managed by the General Inspectorate of Social Security using the national identification number. Pseudonymised details on employer, school and school class identifiers were retrieved from this database. This analysis pertains to all nationally reported cases, which were identified as students and teachers in public schools from this national database linkage occurring between May 4th and July 25th to allow for detecting secondary cases which might have occurred in school settings open until July 15th. Cases from the general working non-teaching population from the same period were assessed using mandatory electronic reporting by the clinical laboratories linked to the national database.

To determine the source of transmission, records of each confirmed case in a primary or secondary school in Luxembourg were reviewed by identifying whether they had contact with other known positives cases in different settings (e.g. family, school, sport, etc). If a contact with a known positive case occurred within an incubation interval of less than 14 days, this case was considered as the probable source. Sources were categorized as family, school, friends or other (sports, multiple probable sources). If there was no reported contact with a positive case, the source was considered unknown. Two independent epidemiologists reviewed complex cases with multiple possible sources.

SARS-CoV-2 incidence rates were calculated by dividing the number of cases in a group by the total number of resident population in that group.

Incidence rate ratios (IRR) were estimated using the *normcdf* function in Matlab.

## Results

### Epidemiological trend analysis

**Fig. 1:**
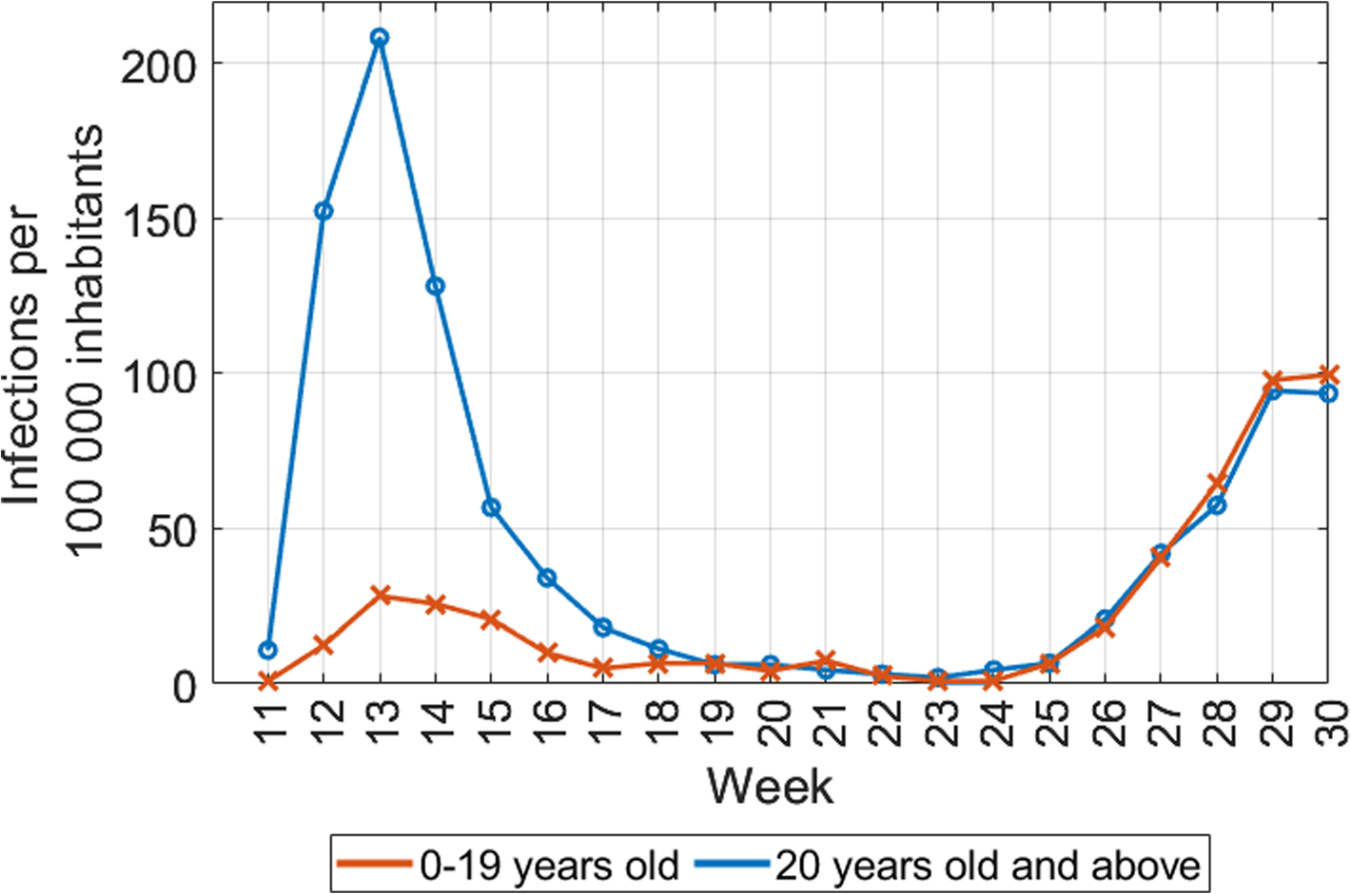
Weekly incidence of SARS-Co-2 infections in Luxembourg between week 11 (March 9–15) and week 30 (July 20–26). Schools closed in week 12 and reopened gradually from week 19 onwards

**Fig. 2:**
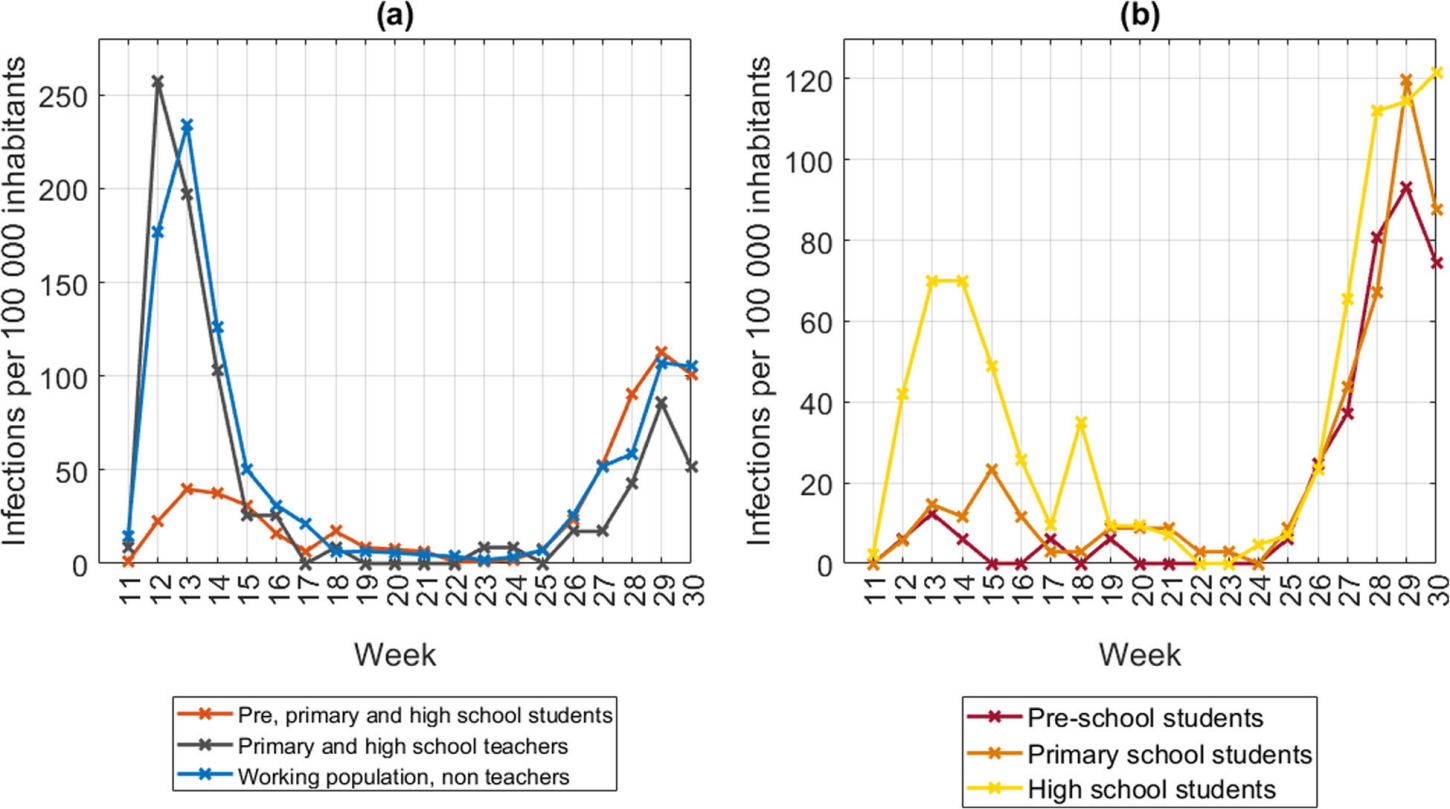
Weekly SARS-CoV-2 incidence in pupils, teachers and the general working population (**a**). Weekly SARS-CoV-2 incidence in students by school level (**b**). Schools closed in week 12 and reopened gradually from week 19 onwards

During the first spring wave in March–April 2020 (weeks 11 to 18), the incidence of SARS-CoV-2 infection in Luxembourg was substantially lower in school-age children compared to the older adult population (see Fig. 1). During the peak (week 13), incidence was 28 per 100,000 in the population aged 0 to 19 compared to 208 per 100,000 for the rest of the population (incidence rate ratio (IRR) 0.13 (95% confidence interval (CI) 0.09–0.19, *p* < 0.001). During the second wave (starting week 25), no differences were observed in the incidence of SARS-CoV-2 infection between school-age children and older adults in the week of July 20–26, IRR 1.06 (95% CI 0.86–1.31, *p* > 0.05). While teachers were primarily affected during the first wave, both teachers and high school pupils were affected during the second wave (Fig. 2).

Incidence rates of SARS-CoV-2 infection in teachers and the general working population were similar during the first wave (weeks 11 to 18), but slightly lower in teachers during the second wave (weeks 25–30) (Fig. 2). Incidence was significantly lower in pupils compared to teachers (IRR 0.20, 95% CI 0.12–0.34, *p* < 0.001) during the first wave, but was higher during the second wave. During week 30, incidence rates in pupils, teachers and general working population were 100, 51 and 105 per 100,000, respectively, leading to an IRR of 0.49 (95% CI 0.21–1.12, *p* > 0.05) between teachers and the general working population and of 0.51 (95% CI 0.22–1.18, *p* > 0.05) between teachers and pupils. Incidence rates were significantly lower in pre-primary school pupils (IRR 0.18, 95% CI 0.04–0.76, *p* < 0.01) and primary school pupils (IRR 0.21, 95% CI 0.08–0.55, *p* < 0.001) than in high school pupils during the first wave, respectively, but differences were less marked during the second wave (IRR 0.61, 95% CI 0.32–1.16, *p* > 0.05 and IRR 0.72, 95% CI 0.46–1.14, *p* > 0.05, respectively).

In the peak week of the first wave, children were 3.44 times less likely to be tested than adults (554 tests and 1909 tests per 100,000 children and adults, respectively) (Supplementary Fig. 1a). In week 30, this ratio was lower at 1.38 (8266 tests and 11,390 tests per 100,000 children and adults, respectively) (Supplementary Fig. 1a). We observed a lower proportion of symptomatic children aged 0 to 19 (54%) compared to adults (69%), likely causing significant numbers of undetected infections in this age group during the first wave. Nevertheless, positivity rates were lower in children (5.1%) than in adults (10.9%), whereas in the second wave they were similar, being 1.2 and 0.8% for children and adults, respectively (Supplementary Fig. 1b). Because of the targeted testing strategy in Luxembourg, teachers benefited from a higher number of tests per 100,000 inhabitants than workers in any other working sectors (174,980 tests per 100,000 for teachers compared to 118,637 tests per 100,000 for the other sectors of activity).

### Transmission in educational settings

Between 4th May and 25th July we identified 390 (92.0%) cases of confirmed SARS-CoV-2 infection in pupils and 34 (8.0%) cases in teachers (Table 1). After excluding cases with no identified source (37.5%), the family or household was the most frequently observed setting of sources of infection (42.5%), followed by school (11.6%) as described hereafter. For 123 (29.0%) cases no data was available in our records to ascertain whether they were present at school, while 73 cases (17.2%) were not present at school (holiday, weekend, restricted class size) posing no infection risk (Table 2). From a total of 228 cases present at school, 150 did not give rise to any secondary cases, while 29 primary cases gave rise to a total of 49 secondary cases (41 pupils and 8 teachers). For the 49 secondary cases, there was no known case in the family and dates between primary and secondary case were compatible with an incubation time of the virus of 2 and 14 days.

**Table 1:** Main characteristics of 424 confirmed cases of COVID-19 in students and teachers in Luxembourg diagnosed between May 4th and July 25th 2020

Characteristics		Number	Percentage
**Sex**	Female	231	54.5%
**Age group (years)**	0–4	16	3.8%
	5–9	91	21.5%
	10–14	115	27.1%
	15–19	113	26.7%
	20–59, student	10	2.4%
	20–59, teacher	34	8.0%
**Status**	Pre- and primary school pupil	176	41.5%
	High school student	214	50.5%
	Primary school teacher	16	3.8%
	High school teacher	18	4.3%
**Probable source**	Family	182	42.9%
	School	49	11.6%
	Friends	16	3.8%
	Other source (e.g. sport) or multiple sources (e.g. family or school)	18	4.2%
	Unknown	159	37.5%
**Symptoms at test**	Asymptomatic	193	45.5%
	Symptomatic	231	54.5%

**Table 2:** Context of transmission of cases by level of education

Context	Preprimary and primary (~ 4–11 years)	Secondary (~ 12–19 years)	Total
**Not present in school**	25 (13.0%)	48 (20.7%)	73 (17.2%)
**Presence unknown**	45 (23.4%)	78 (33.6%)	123 (29.0%)
**Primary case without secondary cases**	88 (45.8%)	62 (26.7%)	150 (35.4%)
**Primary case with secondary cases**	14 (7.3%)	15 (6.5%)	29 (6.8%)
**Secondary cases**	20 (10.4%)	29 (12.5%)	49 (11.6%)
**Total**	192 (100%)	232 (100%)	424 (100%)
**Effective reproductive rate** ^a^	0.20	0.38	0.27

^a^secondary cases / total number of primary cases

Of the 49 within school transmissions, 38 (78%) were pupil-to-pupil within the same class, seven (14%) were teacher-to-pupil, three (6%) were pupil-to-teacher and one was teacher-to-teacher transmission.

In total, 179 positive cases (both pupils and teachers) were estimated to have transmitted SARS-CoV-2 infection to 49 secondary cases, which corresponded to an effective reproductive rate of 0.27 when considering only the school setting. The difference in reproductive rate (ratio of secondary cases to primary cases) did not reach statistical significance (Fisher’s exact test *p* = 0.053) between primary schools (0.2 or 20/102) compared to secondary schools (0.36 or 29/77), but was significantly different (*p* = 0.022) between symptomatic (0.37 or 36/98) compared to asymptomatic primary cases (0.16 or 13/81).

The number of secondary cases per index case varied from 1 to 5 cases at most. No substantial transmission chain or outbreak was identified within schools. It should be noted that of the 49 secondary cases, 40 (81.6%) were under quarantine when they were tested, indicating why transmission was rapidly interrupted. Among the 49 secondary cases, a further total of 12 tertiary transmissions primarily in family members were also identified.

As a result of the contact tracing and quarantine policy in Luxembourg, the 424 cases at schools yielded a total of 2721 contacts placed under quarantine. Of the 726 of those who were students, the average duration of the quarantine was 4.3 days, resulting in a total of 3100 days of quarantine of potentially missed teaching time (not accounting for weekend days). The proportion of people quarantined who became positive depended on the setting of the contact: the risk of transmission was much higher in the family context (14.0%) compared to between friends (5.0%) and, importantly, in school either as a pupil (2.2%) or a teacher (1.1%).

## Discussion

Our investigation concurs with the current view that COVID-19 outbreaks in educational settings appear uncommon [6, 8–12] and that the incidence in educational settings is correlated with the incidence in the general population [6, 10, 13, 14]. However, it should be noted that the limited secondary transmissions observed in Luxembourg occurred in a context with extensive access to testing and contact tracing. This requires a close collaboration of the contact tracing team with the Ministry of Education to rapidly identify and quarantine pupils and teaching staff who had high risk contact with cases and provide access to systematic testing on day 5.

Reasons for the observed differing incidence of SARS-CoV-2 infections between children and adults in the first and second waves Luxembourg could be different exposure levels (because of school closures/opening), different adherence to protective measures and social distancing, different testing strategies and differing proportions of symptomatic infections in these age groups. In the first wave during March–April, due to lower laboratory testing capacity, testing was limited to cases presenting symptoms of COVID-19. As young children are less likely to present symptoms [1], they were generally less likely to be tested. As schools re-opened in May and confirmed cases started to occur in school children, contacts of cases in schools were systematically requested to undergo a PCR test on day 5 after contact regardless of symptoms, thus increasing the general level of testing in children. Thus, it is likely that a combination of these factors (lower exposure during first wave as schools were closed, higher testing during second wave due to mass testing and contact tracing) have contributed to the observed incidence difference in children.

A further aspect of this study suggests that the general lockdown in March–April had a significant impact on the size and duration of the first wave. Previous work has shown the number of reported contacts in the general adult population had increased by 120% during the second period showing an increased potential for COVID-19 spread in early summer [15]. Unfortunately, as the study was limited to adults mainly, it did not investigate whether contact patterns or adherence to personal protective measures and social distancing differed between children and adults.

Our analysis provides valuable additional data on the role of schools [16] in addition to previous studies focusing on outbreaks where current standards of social distancing measures were not or not rigorously adhered to [17–20]. Our findings of limited transmission potential in schools conditional on adequate measures being in place are consistent with other recent studies from Australia, Germany, Ireland, Israel, Norway, the United Kingdom and the United States [10, 21–27]. Our findings differ from those showing that staff-to-staff transmission was more common than student-to-student transmission [10]. While some of these differences could be due to study designs (outbreak vs. routine case ascertainment), they could be also related to recommendations for social distancing between study settings, which are difficult to measure.

One of the major strengths of our study is that it is based on comprehensive national data at a country level which includes all confirmed cases of SARS-CoV-2 as reported by laboratories, their high-risk contacts and exposure at schools. Limitations of our study include that parts of our retrospective analysis are based on administrative contact tracing data, whose primary purpose was to put persons at risk into quarantine, rather than conduct a prospective research study. Detailed exposure information whether cases were present in school prior to becoming a confirmed case was not always recorded and was missing from about a third of cases. Another limitation of our study is that incidence of SARS-CoV-2 in the general population during the summer period was rather limited and it is difficult to extrapolate our findings to other settings or periods with much higher incidence levels in the general population.

To conclude, schools were not a major focus of COVID-19 transmission in Luxembourg during an early summer wave in 2020. Our findings suggest that in a general context of moderate COVID-19 incidence, current prevention measures in schools applied in combination with easy access to testing, isolation and systematic quarantine of class mates, transmission events in schools may be limited in scope. Whether these same measures are sufficient to limit the spread of more transmissible SARS-CoV-2 variants [28] remains to be determined.

## Supplementary Information

**Additional file 1: Supplementary Fig. 1**. Weekly SARS-CoV-2 test rate (a) and positivity rate (b) by age group in residents in Luxembourg. Schools closed in week 12 and reopened gradually from week 19 onwards.

## Data Availability

The datasets analyzed during the current study are available from the corresponding author on reasonable request. Administrative permission to access the database Care+ and Techcare closed to public access was provided by the Health Directorate in Luxembourg.

## Abbreviations

SARS-CoV-2: Severe acute respiratory syndrome coronavirus 2
COVID-19: Coronavirus disease 2019
PCR: Polymerase chain reaction
IRR: Incidence rate ratio
