# Supplementary material for: SARS-CoV-2 transmission in educational settings during an early summer epidemic wave in Luxembourg, 2020
Source: BMC Infect Dis. 2021 May 4;21:417. doi: 10.1186/s12879-021-06089-5 (PMC8093902; doi:10.1186/s12879-021-06089-5)
Supplement: Supplementary file 1 — Additional file 1: Supplementary Fig. 1. Weekly SARS-CoV-2 test rate (a) and positivity rate (b) by age group in residents in Luxembourg. Schools closed in week 12 and reopened gradually from week 19 onwards. [file 12879_2021_6089_MOESM1_ESM.docx]

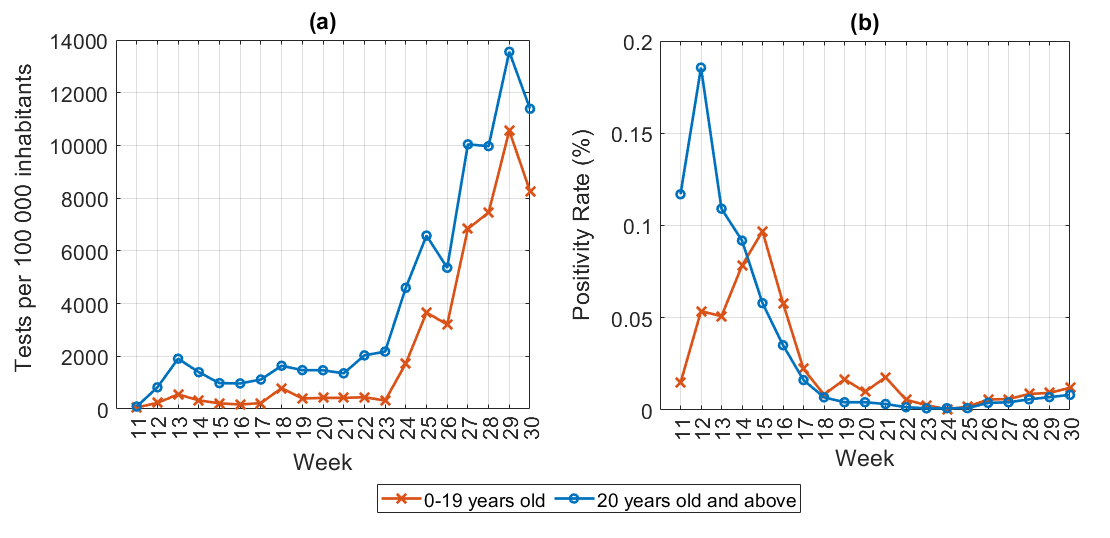


**Supplementary Figure 1**. Weekly SARS-CoV-2 test rate (a) and positivity rate (b) by age group in residents in Luxembourg. Schools closed in week 12 and reopened gradually from week 19 onwards.
